# Preparation of Cationic Amphiphilic Nanoparticles with Modified Chitosan Derivatives for Doxorubicin Delivery

**DOI:** 10.3390/ma14227010

**Published:** 2021-11-19

**Authors:** Xiudong Liu, Huofei Zhou, Weiting Yu, Xin Xiong, Rumen Krastev, Xiaojun Ma

**Affiliations:** 1 College of Environment and Chemical Engineering, Dalian University, Dalian 116622, China; 2 Laboratory of Biomedical Material Engineering, Dalian Institute of Chemical Physics, Chinese Academy of Sciences, Dalian 116023, China; hfzhou@dicp.ac.cn (H.Z.); maxj@dicp.ac.cn (X.M.); 3 The Affiliated Zhongshan Hospital, Dalian University, Dalian 116001, China; 4 NMI Natural and Medical Sciences Institute, University of Tübingen, 72770 Reutlingen, Germany; xin.xiong@nmi.de (X.X.); rumen.krastev@reutlingen-university.de (R.K.); 5 School of Applied Chemistry, Reutlingen University, 72762 Reutlingen, Germany

**Keywords:** cationic amphiphilic chitosan, self-assembled nanoparticles, doxorubicin, drug delivery

## Abstract

Polymeric micelle-like nanoparticles have demonstrated effectiveness for the delivery of some poorly soluble or hydrophobic anticancer drugs. In this study, a hydrophobic moiety, deoxycholic acid (DCA) was first bonded on a polysaccharide, chitosan (CS), for the preparation of amphiphilic chitosan (CS-DCA), which was further modified with a cationic glycidyltrimethylammounium chloride (GTMAC) to form a novel soluble chitosan derivative (HT-CS-DCA). The cationic amphiphilic HT-CS-DCA was easily self-assembled to micelle-like nanoparticles about 200 nm with narrow size distribution (PDI 0.08–0.18). The zeta potential of nanoparticles was in the range of 14 to 24 mV, indicating higher positive charges. Then, doxorubicin (DOX), an anticancer drug with poor solubility, was entrapped into HT-CS-DCA nanoparticles. The DOX release test was performed in PBS (pH 7.4) at 37 °C, and the results showed that there was no significant burst release in the first two hours, and the cumulative release increased steadily and slowly in the following hours. HT-CS-DCA nanoparticles loaded with DOX could easily enter into MCF-7 cells, as observed by a confocal microscope. As a result, DOX-loaded HT-CS-DCA nanoparticles demonstrated a significant inhibition activity on MCF-7 growth without obvious cellular toxicity in comparison with blank nanoparticles. Therefore, the anticancer efficacy of these cationic HT-CS-DCA nanoparticles showed great promise for the delivery of DOX in cancer therapy.

## 1. Introduction

Most anticancer drugs are poorly soluble, which gives rise to considerable challenges for their clinical application. To promote the solubility of drugs, some surfactants such as Cremophor EL/ethanol, and Tween 80 are usually used. These reagents, however, can cause several side effects on the liver and kidneys, and cause dose-dependent hemolysis and acute hypersensitivity reactions [1]. Therefore, numerous nanocarriers for the delivery of poorly soluble or hydrophobic drugs have been developed, such as polymeric nanoparticles, liposomes, hydrogels, and mesoporous silica nanoparticles. Many of them have been engineered for improving drug solubility and stability in serum, enhancing bioavailability in vivo and reducing drug toxicity and side effects in recent decades [2,3,4,5]. Polymeric micelle-like nanoparticles self-assembled from biodegradable and biocompatible amphiphilic block copolymers and hydrophobically modified water-soluble polymers have been extensively investigated for drug delivery application [6,7,8]. The cationic polymeric nanoparticles usually display good water solubility and high cellular uptake efficiency [9]. Moreover, cationic polymeric nanoparticles are usually made up of hydrophobic core and hydrophilic cationic shell based on self-assembly. The hydrophobic core can serve as a drug reservoir for most hydrophobic anticancer drugs, and the cationic shell can interact with DNA, siRNA, protein, or peptide to accomplish simultaneously co-delivery of different kinds of drugs on chemotherapy with synergistic effects [10,11,12,13].

Chitosan (CS), the only natural cationic polysaccharide, has been widely used for the delivery of both anticancer drugs and biochemical drugs [14,15,16,17,18,19,20,21,22] in the above-mentioned forms. In our previous study, CS was modified with a hydrophobic molecule of deoxycholic acid and hydrophilic glycidyl to obtain soluble amphiphilic chitosan derivative (G-CS-DCA), which showed effective delivery of doxorubicin (DOX) and anticancer activity in MCF-7 cells [18]. However, the zeta potential values were as small as 0–2 mV, which was considered insufficient for the improvement of cell absorption of nanoparticles. In this study, CS was first modified with a hydrophobic molecule of deoxycholic acid (DCA) for the preparation of amphiphilic chitosan (CS-DCA). Then, a cationic hydrophilic molecule glycidyltrimethylammounium chloride (GTMAC) was grafted on CS to produce a novel cationic amphiphilic chitosan derivative, N-(2-hydroxy)propyl-3-trimethylammonium CS-DCA (HT-CS-DCA), with the purpose to improve both the solubility and the positive charge density. The grafting of hydrophobic and hydrophilic moieties on CS forming HT-CS-DCA was demonstrated by Fourier transform infrared (FTIR) spectroscopy and ^1^H NMR. The elemental analysis was used to calculate the degree of substitution. The fluorescence probe, pyrene, was used to prove the formation of micelle-like HT-CS-DCA nanoparticles. The morphology, size, and zeta potential of nanoparticles were tested. Then, doxorubicin (DOX) was loaded into HT-CS-DCA nanoparticles, and the DOX release profile was studied. The growth inhibition against MCF-7 cells of HT-CS-DCA nanoparticles and antitumor efficacy of DOX-loaded HT-CS-DCA nanoparticles was evaluated through methyl tetrazolium (MTT) assay and confocal microscopy, which confirmed the low toxicity of carrier but a high cellular uptake and significant antitumor activity of DOX-loaded HT-CS-DCA nanoparticles.

## 2. Materials and Methods

### 2.1. Materials

The raw chitosan was bought from Yuhuan Ocean Biochemical Co., Ltd. (Zhejiang, China) with M_w_ = 230 kDa, degree of deacetylation, DD = 97%. Both 1-ethyl-3-(3-dimethylaminopropyl) carbodiimide hydrochloride (EDC•HCl), N-hydroxysuccimide (NHS), and fluorescent pyrene were bought from Acros Organics (Geel, Belgium). Deoxycholic acid (DCA), and glycidyltrimethylammounium chloride (GTMAC) were obtained from Sigma Co. (Cream Ridge, NJ, USA). Doxorubicin•HCl (DOX•HCl) was a gift from Hisun Pharmaceutical Co. Ltd., (Zhejiang, China). All other chemicals and materials were obtained from Damao chemical reagent Co., (Tianjing, China) and applied as received.

### 2.2. Preparation of Cationic Amphiphilic Chitosan HT-CS-DCA

A total of 24 kDa CS was obtained with the degradation from raw CS using NaNO_2_ [23]. The introduction of hydrophobic molecule DCA with the help of EDC and NHS resulted in amphiphilic chitosan derivative CS-DCA. Briefly, DCA (at a feed ratio of 10% to 30% of deoxycholic acid to glucosamine units), activators EDC and NHS (EDC/DCA = 1.2/1) were dissolved in 100 mL of DMSO and pre-reacted for 30 min; both DCA and EDC/NHS were mixed for activation. Subsequently, the activated DCA solution was dropped into CS solution (4 g 24 kDa CS dissolved in 200 mL acidic solution (pH 5.6)) in 60 min. After reaction for 24 h, the solution was added into methanol/NaOH solution (300 mL, pH 8). With the centrifugation, the sediment was obtained, washed with a mixture of alcohol and water, respectively, and then vacuum dried. CS-DCA samples with different degrees of substitute (DS) of DCA were obtained by changing the amounts of DCA.

Secondly, a cationic hydrophilic molecule glycidyltrimethylammounium chloride (GTMAC) was grafted on HCS-DCA to produce a novel cationic amphiphilic chitosan derivative (HT-CS-DCA), with the purpose to improve both the solubility and the positive charge density. CS-DCA (1.0 g) was dispersed in 50 mL acetic acid solution for at least 6 h. Then, GTMAC was added dropwise at the mole ratio of 3:1–5:1 (GTMAC to glucosamine units in CS-DCA) to produce HT-CS-DCA with different DS of GTMAC. After reaction for 24 h at 1000 rpm at 50 °C, NaOH was used to change the solution pH to 8. The mixture was separated from undissolved polymer with centrifugation (8000 rpm, 30 min). Finally, the solution was dialyzed against deionized water for more than 3 days, with repeated changes of water and lyophilized for further experiments.

### 2.3. Preparation HT-CS-DCA and DOX-Loaded HT-CS-DCA Nanoparticles

A total of 10 mL of PBS (0.02 M, pH 7.4) was used to dissolve HT-CS-DCA (20 mg). The solution was placed into a probe sonicator (JY92-II, Ningbo, Zhejiang, China) and sonicated at 60 W for 2 min with a pulse off for 1 s and an interval of 5 s. Then, the membrane filter (pore size: 0.45 μm, Millipore) was used to remove possible impurities or too large particles. The obtained HT-CS-DCA nanoparticles solution was stored for further test.

DOX was encapsulated in HT-CS-DCA nanoparticles by dialysis. For this procedure, 4 mL water/DMSO (*v*/*v* = 1/9) mixture was used as a solvent for HT-CS-DCA (20 mg), while DOX was neutralized by a 3 molar excess of triethylamine in 1 mL of DMSO. After mixing DOX and HT-CS-DCA solutions, the mixture was stirred for one hour and then dialyzed against PBS (0.01 M, pH 7.4) using a dialysis membrane (MWCO = 3500 Da) for 24 h at room temperature. Finally, the dialyzed solution was filtered through a 0.8 μm membrane and freeze dried. All procedures were performed in the dark.

### 2.4. Measurements

#### 2.4.1. Characterization of Chitosan Derivatives

The synthesized CS-DCA and HT-CS-DCA were firstly dried and characterized with a Bruker spectrometer (VECTOR22, Fällanden, Switzerland). The operating condition was from 4000 to 400 cm^−1^ with a resolution of 4 cm^−1^.

Using a Bruker Avance 500 MHz spectrometer (Fällanden, Switzerland), the above samples were tested to give ^1^H NMR spectra in deuterated water (D_2_O) at room temperature.

The DS of DCA or GTMAC was referred to as the percentage of DCA or GTMAC in 100 chitosan residues. The C, H, and, N amounts of each sample were determined by elemental analysis (Elemental Analyzer Vario EL, III, Hanau, Germany). Thereafter, the DS of DCA or GTMAC of the synthesized CS samples was calculated according to the C, H, and N ratios in CS and its derivatives. The derivatives were designated as HT_(DS of GTMAC)_-CS-DCA_(DS of DCA)_.

#### 2.4.2. Critical Aggregation Concentration (CAC)

To determine the critical aggregation concentration (CAC) of HT-CS-DCA samples in PBS buffer (0.02 M, pH 7.4), pyrene was used as a fluorescence probe. In volumetric flasks, 5 mL HT-CS-DCA solution with different concentrations was mixed with pyrene (final concentration of 6.0 × 10^−7^ M). A luminescence spectrometer (LS55, PerkinElmer, Waltham, MA, USA) was used to give fluorescence spectra at room temperature. The emission spectra were collected at a scan speed of 120 nm/min within the wavelength range of 350 to 450 nm and the excitation wavelength was 336 nm. The first band (373 nm, I_1_) and the third band (383 nm, I_3_) in the emission spectra were obtained, then the intensity ratio (I_1_/I_3_) was calculated with the change of polymer concentrations. Furthermore, the first turning point in the curve of I_1_/I_3_ ratio−polymer concentration was considered as the CAC.

#### 2.4.3. Morphology, Size, and Zeta Potential

The morphology of HT-CS-DCA nanoparticles was observed by TEM (JEM−2000 EX, Joel Ltd., Tokyo, Japan), operating at an acceleration voltage of 120 kV.

A Malvern analyzer for nanoparticles (Zetasizer Nano ZS90, Malvern, UK) was applied to determine the nanoparticle size. At least three independent measurements for each nanoparticle (2 mg/mL) were carried out, and the results regarding size and zeta potential were displayed as the average value ± standard deviation.

#### 2.4.4. Drug Loading

The loading amount of DOX in HT-CS-DCA nanoparticles was analyzed by UV–Vis spectrophotometer (Shimadzu Co., Kyoto, Japan) with the predetermined calibration curve (a serial dilution of DOX in PBS solutions at 481 nm). The drug loading content (LC) of DOX was calculated according to Equation (1).

(1) $$LC(wt\%)=\frac{\mathrm{drug}\;\mathrm{amount}\;\mathrm{in}\;\mathrm{nanoparticles}}{\mathrm{amount}\;\mathrm{of}\;\mathrm{nanoparticles}}\times 100\%$$

### 2.5. Release of DOX from HT-CS-DCA Nanoparticles In Vitro

DOX-loaded HT-CS-DCA nanoparticles were put into PBS (0.02 M, pH7.4), and the release behavior was studied in vitro. Briefly, DOX−HT-CS-DCA nanoparticles (3 mg) were dispersed in 3 mL PBS (pH 7.4), then the suspension was transferred to a dialysis membrane (MWCO = 3500 Da). The release was achieved in the dialysis membrane by gentle agitation (100 rpm) in 30 mL PBS at 37 °C. A total of 3 mL of samples was extracted from the outer solution at fixed times, and 3 mL fresh buffer was added. According to the standard curve, DOX concentrations could be determined.

### 2.6. Confocal Laser Scanning Microscopic (CLSM) Observations on Cellular Uptake

Cellular uptake by MCF-7 human breast cancer cells and intracellular release of DOX from HT-CS-DCA nanoparticles were examined using CLSM (Leica, Germany). MCF-7 cells were introduced onto plates with an initial cell density of 2 × 10^4^ cells/well, cultivated with media containing 10% FBS for 24 h. Then, the DOX-HT_42_-CS-DCA_3.2_ nanoparticles or free DOX were dissolved in culture media at a concentration of 5 µg/mL. The cells were exposed to the media with either DOX-HT_42_-CS-DCA_3.2_ nanoparticles or DOX•HCl for 2 and 5 h, respectively, at 37 °C in a humidified environment with 5% CO_2_. After removing the culture media, the MCF-7 cells were washed with PBS three times prior to the CLSM-imaging with excitation wavelength 485 nm and emission wavelength 595 nm.

### 2.7. Cellular Viability Study In Vitro

MCF-7 cells were seeded in the 96−well plate (10,000 cells/well), and cultured overnight, with 200 µL of RPMI 1640 (10% FBS) in a humidified environment at 37 °C with 5% CO_2_ to assess the cytotoxicity of CS derivatives and DOX-loaded nanoparticles. Meanwhile, samples of different drug formulations were added in PBS buffer (pH 7.4), followed by serial dilution to obtain a range of final drug concentrations. At a suitable time, a total 200 µL mixture (20 µL of nanoparticle solution and 180 µL of fresh medium) was used to replace the old culture medium in plates to keep the DOX concentration within a certain range. Finally, the cytotoxicity of CS derivatives was also determined in the culture medium (0–1000 µg/mL). The cells were cultured under the same condition as described above for 48 h. Thereafter, aliquots of assay solution containing 180 µL fresh medium and 20 µL MTT solutions (5 mg/mL) were applied to replace the mixture in each well. After 4 h of incubation, the assay solution was removed and 200 µL DMSO was added to each well. Then, the absorbance of the dissolved formazan crystals was measured by a microplate reader (Well Scan MK3, Labsystems Dragon, Finland) at 50 nm, and the reference wavelength was 630 nm. The absolute absorbance of each well was then calculated by subtraction of -absorbance_630nm_ from the—absorbance_550nm_. Then, the relative cell viability can be calculated using Equation (2).

(2) $$\mathrm{cell}\;\mathrm{viability}(\%)=\frac{{\mathrm{OD}}_{\mathrm{sample}}\;}{{\mathrm{OD}}_{\mathrm{control}}}\times 100\%$$

where OD_control_ was determined without polymers, and OD_sample_ was determined with polymers. The data were displayed as average value ± SD for six parallel samples.

## 3. Results and Discussion

### 3.1. Preparation of Cationic Amphiphilic Chitosan Derivatives HT-CS-DCA

**Scheme 1:**
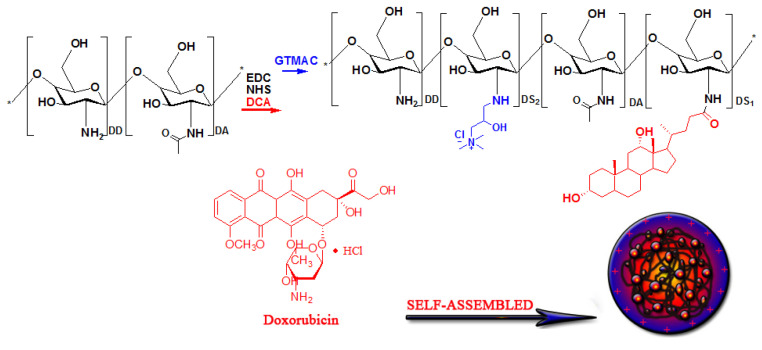
The synthesis route of HT-CS-DCA and schematic of encapsulation of DOX based on self-assembly.

Scheme 1 displayed the route of synthesizing cationic amphiphilic chitosan derivatives HT-CS-DCA.

**Figure 1:**
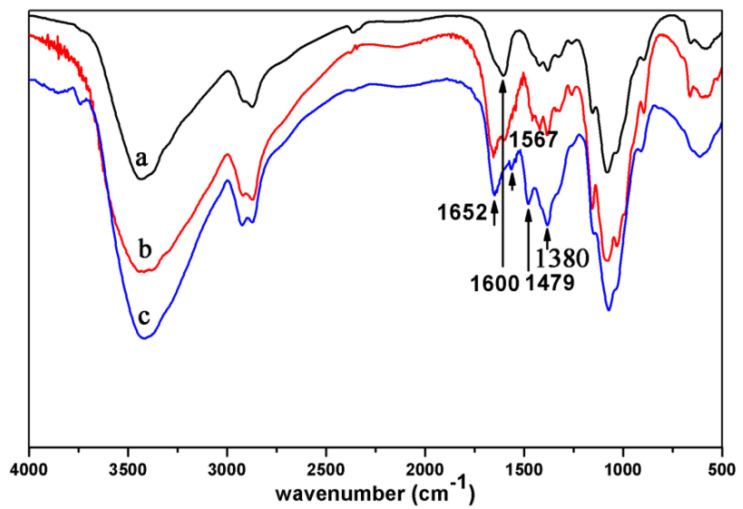
The FTIR spectra of (**a**) CS (DD = 97%); (**b**) CS-DCA_3.2_; (**c**) HT_42_-CS-DCA_3.2_; 42 and 3.2 refer to the DS of GTMAC and DCA, respectively.

With EDC and NHS, hydrophobic DCA was first grafted onto chitosan−producing amphiphilic derivative CS-DCA. In FTIR analyses, a clearly enhanced peak at 1652 cm^−1^ (amide I band) in CS-DCA_3.2_ (DS of DCA is 3.2) can be seen compared with the CS spectrum. The peak at 1600 cm^−1^ (−NH_2_) demonstrated a weakened N−H bending vibration (Figure 1). It was found that the amide I band (1652 cm^−1^) sharpened as the feed ratio of DCA to chitosan increased [17]. By elemental analysis, the DS of DCA can be calculated as above−mentioned.

Secondly, GTMAC was grafted on CS-DCA to obtain HT-CS-DCA following the synthesis route in Scheme 1. The FTIR spectra of CS, CS-DCA_3.2_, and HT_42_-CS-DCA_3.2_ (DS of GTMAC is 42) are shown in Figure 1. By comparing the FTIR spectra of CS-DCA_3.2_ and HT_42_-CS-DCA_3.2_, the peak 1600 cm^−1^ (−NH_2_) further weakened, and the peak 1567 cm^−1^ was attributed to the vibration of amide II of GTMAC. Meanwhile, the increase in peaks at 1479 cm^−1^ (C−H deformations) and 1380 cm^−1^ (−CH_3_ symmetrical deformation) also meant the successful grating of GTMAC on CS-DCA.

**Figure 2:**
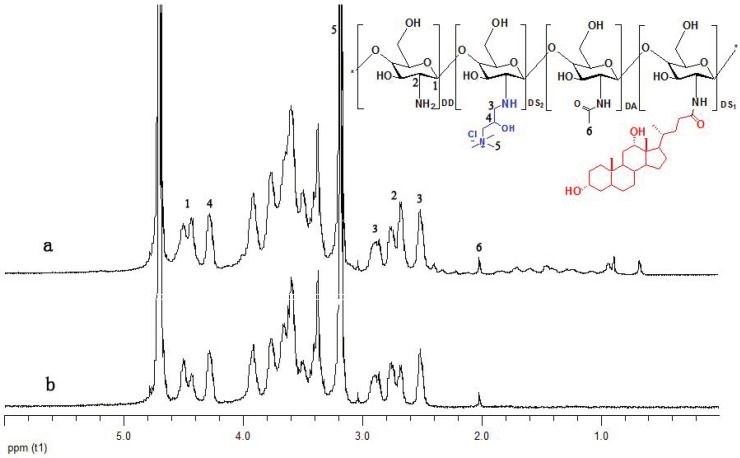
Spectra of chitosan derivatives analyzed by ^1^H NMR: (**a**) HT_42_-CS-DCA_4.2_; (**b**) HT-CS.

Figure 2 shows the ^1^H NMR spectra of HT-CS and HT_42_-CS-DCA_4.2_. The peak at 3.2 ppm represented −^+^N(CH_3_)_3_ in GTMAC, and the peaks at 2.9 ppm and 2.5 ppm showed the −NH−CH_2_− group in GTMAC. The peaks at 2.67–2.76 ppm belonged to C−2 in the saccharide unit for both HT-CS and HT-CS-DCA. Based on the comparison of ^1^HNMR spectra of Figure 2a,b, it might be that the hydrophobic molecule DCA was introduced into CS resulting in new peaks between 0.7 and 1.9 ppm.

### 3.2. Morphology, CAC, Size, and Zeta Potential of HT-CS-DCA Nanoparticles

**Figure 3:**
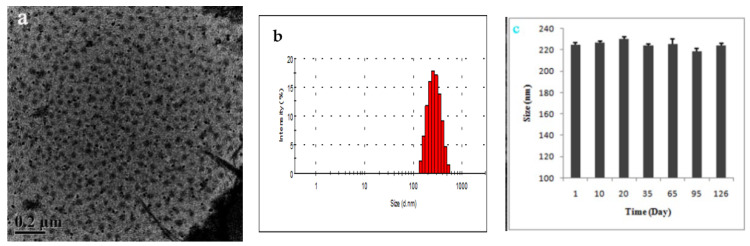
TEM images (**a**), size distribution (**b**), and size stability with time (**c**) of cationic amphiphilic HT_42_-CS-DCA_3.2_ nanoparticles in PBS (2 mg/mL) at room temperature.

TEM characterization demonstrated that the HT-CS-DCA nanoparticles have spherical shapes (Figure 3a). Together with the dynamic light scattering detection (Figure 3b), HT-CS-DCA nanoparticles were displayed relatively uniform in size. Moreover, the nanoparticles maintained higher size stability when stored at room temperature for more than 4 months (Figure 3c).

On the one hand, the CAC value is considered as an index to demonstrate the formation of micelle−like nanoparticles by self−assembly; on the other hand, it is an indication for nanoparticle stability in the circulatory system after the drug administration. As a molecular fluorescence probe, pyrene is useful to test the CAC of amphiphilic polymers. The effect of different DS values of DCA and GTMAC on the CAC of HT-CS-DCA is displayed in Table 1. With the DS increase in hydrophobic DCA and the DS decrease in hydrophilic GTMAC, CAC values of HT-CS-DCA reduced. Moreover, CAC values ranging from 0.023 to 0.079 mg/mL depended on DS of DCA (2.3 to 4.2) and GTMAC (42 to 84). It suggested that stable HT-CS-DCA nanoparticles were formed at low concentrations.

To avoid being recognized, caught, and destroyed by the reticuloendothelial system, it is necessary to control the particles size as small as possible to obtain long circulation [24]. The size and zeta potential of HT-CS-DCA nanoparticles ranged from 180 to 240 nm and 14–24 mV with the change in DS of DCA from 2.3 to 4.2 and DS of GTMAC from 42 to 84 (Table 1). The positive zeta potential of HT-CS-DCA nanoparticles may be beneficial for their high cellular uptake efficiency. With increasing DS of DCA to the same extent, the size and zeta potential increased possibly due to the higher self−aggregation capability between inter− and intra−amphiphilic chitosan molecules with higher hydrophobic interaction. Additionally, the increase in positive charge also drove inter- and intra−chain repulsion of chitosan molecules, leading to larger particles.

### 3.3. Release of DOX from HT-CS-DCA Nanoparticles In Vitro

The above CAC test displayed that poor soluble or hydrophobic drugs similar to hydrophobic pyrene can be encapsulated into HT-CS-DCA nanoparticles. In this study, DOX with poor solubility was loaded into HT_42_-CS-DCA nanoparticles by dialysis of polymer/DOX in DMSO/water solution against water. When the DS of DCA increased from 2.3 to 4.2, the drug loading (LC) increased from 4.4% to 6.1% under the condition that the theoretical drug loading content is 10%. Correspondingly, the size of DOX-loaded HT_42_-CS-DCA nanoparticles was 30−60 nm larger than that of empty ones, while the PDI almost kept monodisperse in PBS.

**Figure 4:**
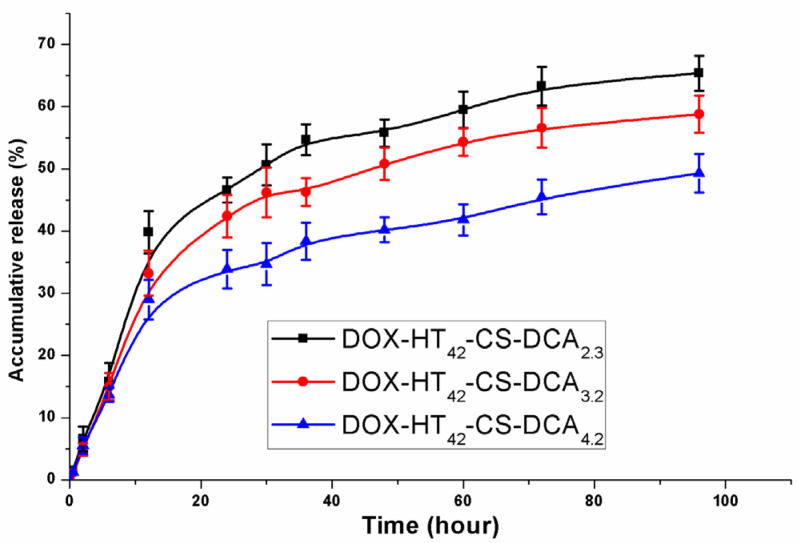
Release profile of DOX from HT_42_-CS-DCAs nanoparticles in 0.02 M PBS (pH 7.4) at 37 °C. Data represent mean ± s.d. (*n* = 3).

With regard to evaluating drug delivery systems, the in vitro release behavior is usually determined, which can provide useful guidance for the in vivo pharmacokinetics of drugs. Furthermore, the ideal drug delivery system for cancer therapy could hold the drug during circulation but release the drug in a target. The in vitro release behavior was evaluated at 37 °C in PBS (pH 7.4). As shown in Figure 4, the accumulative amount of DOX from HT_42_-CS-DCA nanoparticles was only 5% in the first 2 h meaning no obvious burst release. Then, the accumulative amount of DOX quickly and steadily increased in 24 h, followed by a slow and flat increase in the next 72 h. Meanwhile, both the release speed and the accumulative amount of DOX decreased with the increase in DS of DCA in the whole release process. For instance, the accumulative amount of DOX from HT_42_-CS-DCA_2.3_ nanoparticles was around 64%, while that from HT_42_-CS-DCA_4.2_ nanoparticles was only 47% at 96 h. It can be ascribed to the higher grafting of hydrophobic DCA, which hindered the release of DOX and suggested that the higher substitution of the hydrophobic moiety is beneficial for the sustained release of poor soluble or hydrophobic drugs.

### 3.4. Confocal Laser Scanning Microscopic (CLSM) Observations on Cellular Uptake

**Figure 5:**
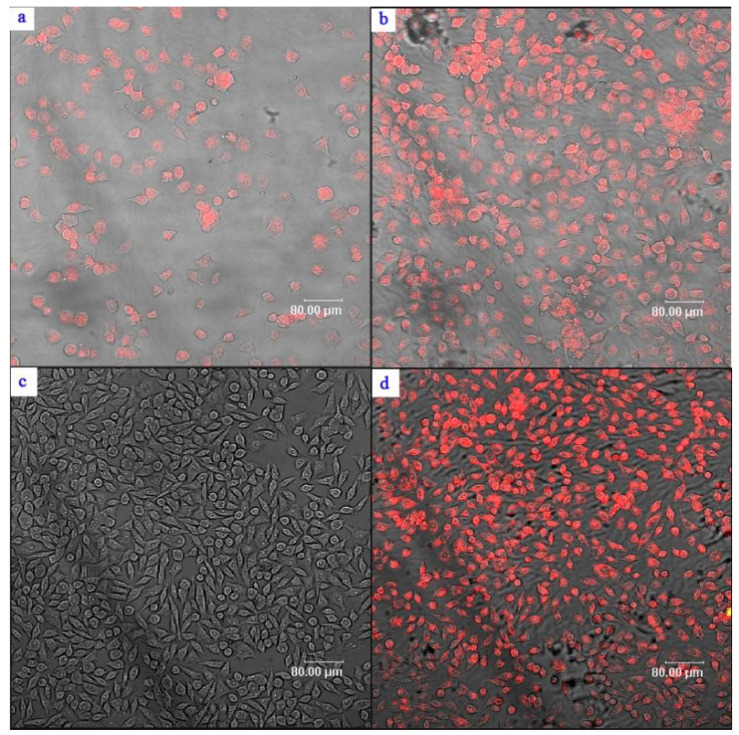
CLSM images of MCF-7 cells cultured with DOX and DOX−HT_42_-CS-DCA_3.2_ nanoparticles (5 µg/mL of DOX): (**a**) free DOX after 2 h incubation; (**b**) free DOX after 5 h incubation; (**c**) DOX−HT_42_-CS-DCA_3.2_ nanoparticles after 2 h incubation; (**d**) DOX−HT_42_-CS-DCA_3.2_ after 5 h incubation.

The cellular uptake of DOX−HT_42_-CS-DCA nanoparticles was detected by CLSM. After incubating MCF-7 cells (Shanghai Institute of Biochemistry and Cell Biology, Chinese Academy of Sciences) with DOX−HT_42_-CS-DCA_3.2_ and free DOX•HCl for 2 and 5 h, the medium was discarded, and the cells in the wells were washed with PBS. As shown in Figure 5, a time−dependent fluorescent intensity was observed within 5 h (Figure 5a,b). Interestingly, after 2 h incubation with free DOX•HCl, a certain degree of fluorescence intensity was observed (Figure 5a); however, no fluorescence intensity was detected while cells were incubated with DOX−HT_42_-CS-DCA_3.2_ nanoparticles (Figure 5c). The results may be ascribed to the self-quenching effect of DOX in nanoparticles—even nanoparticles entered cells, and fluorescence only could be detected when DOX is released [25,26]. There was an increase in fluorescence intensity observed after cell incubation with DOX−HT_42_-CS-DCA_3.2_ nanoparticles for 5 h (Figure 5d). These results were in line with the release profile of DOX from DOX−HT_42_-CS-DCA_3.2_ nanoparticles in vitro because about 15% of DOX was released at this time. Notably, Figure 5b indicates that free DOX•HCl distributed in the cell nucleus, while the fluorescence of DOX−HT_42_-CS-DCA_3.2_ nanoparticles was mainly dotted in the cytoplasm and partly entered in the cell nucleus (Figure 5d). It was shown that cationic DOX−HT_42_-CS-DCA_3.2_ nanoparticles were taken up by cells through endocytic vesicles, then escaped from the endosome and/or the lysosome for entering the cytoplasm, accompanied by the cytosolic release of DOX. Many unprotonated 1°−, 2°−amines in chitosan derivatives will show different pKa values with the crowded amines and will provide a proton buffering effect in a pH range [27]. The pH of the endosomes is lower, the proton buffering capacity of chitosan derivatives correlate with 4°-amines from GTMAC, and is in favor of influx of protons, chloride ions, and water into the endosomes, which give rise to swelling and burst of endosome due to the increase in osmotic pressure.

### 3.5. Cellular Viability Study In Vitro

**Figure 6:**
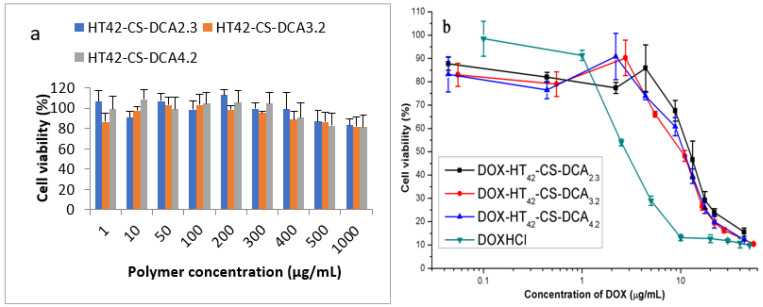
Cytoactivity test of MCF-7 cells cultured with HT_42_-CS-DCA nanoparticles for 48 h (**a**); the results represent the average value ± SD (*n* = 6). Cytoactivity test of MCF-7 cells cultured with DOX−HT_42_-CS-DCA nanoparticles and DOX•HCl for 48 h (**b**); the results represent the average value ± SD (*n* = 6).

The MCF-7 cell line was employed to investigate the anticancer efficacy of DOX−HT_42_-CS-DCA_3.2_ nanoparticles and free DOX•HCl. In order to demonstrate that DOX released from chitosan derivatives nanoparticles, instead of HT_42_-CS-DCA itself, kill the cells, H_42_-CS-DCA nanoparticles without drugs were added in cells as a control group. The results revealed that these nanoparticles have low cytotoxicity because more than 80% of cells were still viable even under the concentration of the H_42_-CS-DCA nanoparticles as high as 1000 µg/mL (Figure 6a). However, as the concentration of DOX in HT_42_-CS-DCA nanoparticles changed from 1 µg/mL to 50 µg/mL, the cell viability of MCF-7 reduced from almost 100% to less than 15% after incubation for 48 h. The free drug dissolved in PBS showed similar anticancer efficacy to DOX−HT_42_-CS-DCA_3.2_ nanoparticles. As shown in Figure 4 and Figure 6b, DOX released slowly from HT_42_-CS-DCA nanoparticles before entering the nucleus to take drugs into effect through interaction with DNA; therefore, the anticancer efficacy of DOX−HT_42_-CS-DCA lagged a little behind free DOX•HCl within 48 h. We observed there was almost no difference in the anticancer efficacy of DOX−HT_42_-CS-DCA nanoparticles with varied DS of DCA, because the anticancer efficacy may be influenced by several factors including particles size, zeta potential, the interaction strength between DOX and DCA, and the site of the drug inside the micelle core.

## 4. Conclusions

We successfully developed a novel cationic soluble amphiphilic chitosan derivatives HT-CS-DCA to fabricate nanocarriers for the delivery of anticancer drugs, doxorubicin. Based on self−assembly, the cationic amphiphilic HT-CS-DCA nanoparticles were spherical in shape, with an average size around 200 nm and zeta potential ranging from 14 to 24 mV in PBS (pH 7.4). The CAC values of HT-CS-DCA nanoparticles were in the range from 0.079 mg/mL to 0.023 mg/mL according to the variation of DS values of DCA and GTMAC. DOX encapsulated in HT-CS-DCA nanoparticles showed no obvious burst release and kept a slow, sustained release, with adjustable release speed by changing DS of DCA. Fluorescence imaging experiments indicated DOX encapsulated in HT-CS-DCA nanoparticles could be easily delivered into tumor cells and then released from nanoparticles to inhibit tumor cell growth. Therefore, HT-CS-DCA nanoparticles have a high potential of delivering DOX and other kinds of poorly soluble or hydrophobic drugs.

## Figures and Tables

**Table 1:** CAC, size, and zeta potential of HT-CS-DCA nanoparticles.

Sample ^a^	CAC (mg/mL)	Size (nm) ^b^	Zeta Potential (mV)
HT_42_-CS-DCA_2.3_	0.079	185.8 ± 3.4 (0.18)	14.1 ± 0.6
HT_42_-CS-DCA_3.2_	0.035	224.0 ± 2.6 (0.09)	14.6 ± 0.4
HT_42_-CS-DCA_4.2_	0.023	233.6 ± 3.0 (0.08)	15.7 ± 0.8
HT_61_-CS-DCA_3.2_	0.045	229.0 ± 1.7 (0.07)	17.1 ± 1.0
HT_84_-CS-DCA_3.2_	0.060	235.2 ± 1.4 (0.08)	23.3 ± 3.2

^a^ HT_42_-CS-DCA_3.2_ shows the DS of GTMAC is 42, DS of DCA is 3.2, respectively. ^b^ Values in parentheses display the polydispersity index (PDI).

## Abbreviations

CS	Chitosan
CLSM	Confocal laser scanning microscopy
CAC	Critical aggregation concentration
DCA	Deoxycholic acid
DOX	Doxorubicin
EE	Encapsulation efficiency
EDC•HCl	1-ethyl-3-(3-dimethylaminopropylCarbodiimide hydrochloride
GTMAC	Glycidyltrimethylammounium chloride
HT-CS-DCA	N-(2-hydroxy)propyl-3-trimethylammnonium-chitosan-deoxycholic acid
LC	Loading content
MCF-7	Human breast cancer cells
MTT	Methyl tetrazolium
MWCO	Molecular weight cut-off
NHS	N-hydroxysuccimide
PDI	Polydispersity index
